# Comparison of Low Cost Miniature Spectrometers for Volcanic SO_2_ Emission Measurements

**DOI:** 10.3390/s90503256

**Published:** 2009-04-28

**Authors:** Euripides P. Kantzas, Andrew J. S. McGonigle, Robert G. Bryant

**Affiliations:** Department of Geography, University of Sheffield, Sheffield, S10 2TN, UK, E-Mails: ggp07epk@shef.ac.uk (E. P. K.); a.mcgonigle@shef.ac.uk (A. J. S. M.); r.g.bryant@shef.ac.uk (R. G. B.)

**Keywords:** ultraviolet spectrometers, volcanic SO_2_ monitoring, differential optical absorption spectroscopy

## Abstract

Miniature ultraviolet USB coupled spectrometers have become ubiquitously applied over the last decade for making volcanic SO_2_ emission rate measurements. The dominantly applied unit has recently been discontinued however, raising the question of which currently available devices should now be implemented. In this paper, we consider, and make recommendations on this matter, by studying a number of inexpensive compact spectrometers in respect of measurement performance and thermal behaviour. Of the studied units, the Avaspec demonstrated the best prospects for the highest time resolution applications, but in the majority of cases, we anticipate users likely preferring the less bulky USB2000+s.

## 1. Introduction

The last decade has seen a rapid proliferation of compact and low cost USB powered spectrometers to the international volcanology community, for use in remotely sensed measurements of volcanic gas plumes. These units have now succeeded the considerably bulkier correlation spectrometers [1], in becoming the standard ground based tool for monitoring volcanic SO_2_ emission rates [2-7]. Data are typically collected by coupling these spectrometers to vertically pointing telescopes, traversing beneath the plume, by car, boat or plane, and recording geo-referenced spectra, from which overhead SO_2_ column amounts are determined, then integrated over the plume cross-section, before multiplication by the plume transport speed to output fluxes. The devices have also been deployed in scanning configurations, whereby the field of view is instead rotated through the plume in an automated manner, using stepper motor mounted fore-optics [8-11]. This provides higher time resolution data (every few minutes), bringing the prospect of non-aliased corroboration between volcanic geophysical (typically ≈ 1 Hz) and geochemical datasets a little closer, with promise for improving our understanding of subterranean magmatic processes, and utility in eruption forecasting.

In addition to SO_2_, the devices have also been used to detect volcanic plume species such as BrO [12, 13]. This is highly significant, as the latter gas is implicated in halogen catalysed ozone depleting reactions, indicating that volcanic plumes could exert important controls upon the troposphere's oxidation capacity. Further, the spectrometers have enabled far more accurate measurements of plume transport speed than available hitherto, via cross correlation of SO_2_ data streams from multiple under plume units, thus significantly reducing error in calculated fluxes [14,15]. Finally, due to the small size and weight of the units (≈ 200g), they are also readily portable, with all required auxiliary components (e.g., miniature laptop computer), for unmanned aerial vehicle measurements opening up new prospects for spectroscopic surveillance of volcanic plumes, with monitoring personnel stationed further away from the hazardous targets [16].

Recently the device dominantly applied in this application, the Ocean Optics USB2000 has been discontinued, prompting the question: which currently available miniature USB coupled spectrometer is now most suitable for volcanologists to use in making SO_2_ flux measurements. Here we report the results of a study aimed at addressing this, in which we inter-compare a number of such devices with the USB2000 in respect of their measurement accuracy, including a discussion on their susceptibility to thermal effects that can induce errors. Whilst our focus is on the volcanic application of these units, this study is also germane to spectroscopic studies of other SO_2_ emission sources, e.g., from power stations and agrochemical plants.

## 2. Instrumentation

Seven spectrometers were investigated in this study: three Ocean Optics (OO) USB2000s (serial numbers: USB2G3709, USB2G1110 and USB2G2385), two OO USB2000+ units (serial numbers: USB2+E001 and USB2+F002), an OO USB4000 (USB4C0004) and an Avantes B.V. Avaspec-2048-USB2 (0802095U1). The latter units were chosen, as they fall within a similar price band (≈ €2,700 at January 2009, including the required software) as the USB2000 did, fitting with the low cost philosophy implicit in the initial adaptation of the USB2000 to volcano monitoring: spectrometers should be suitable for widespread dissemination to countries where risks are high, yet monitoring budgets are low.

**Figure 1.**
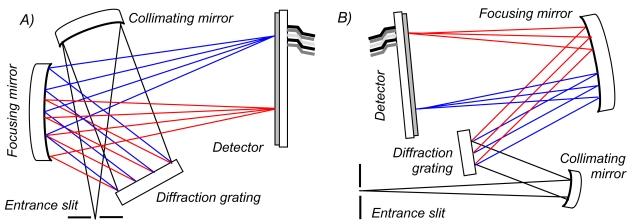
Schematic showing the operating principles of the spectrometers discussed here. Light enters the spectrometer through the entrance slit, is collimated by a curved mirror, then segregated into constituent wavelengths (e.g., blue and red) by a diffraction grating. These spectral components are then imaged onto discrete pixels of a CCD array detector, by a second curved mirror, so to obtain a spectrum. Both folded (A; e.g., OO) and non-crossed (B; e.g., Avaspec) configurations are shown. The figures are not to scale.

The basic operating principle of these units (Figure 1) is that the light enters the spectrometer through an entrance slit (50 × 1000 μm in each case, providing equivalent input intensity). This divergent light is then collimated by a curved mirror, and dispersed into its spectral components by a diffraction grating. Each of these wavelengths is reflected at a different angle, from the latter component, and is then imaged onto discrete pixels of a linear silicon CCD array using another concave mirror. By reading the outputs of this detector, a spectrum is obtained, therefore. In the case of the OO spectrometers, the configuration is crossed, e.g., the light paths between the optical components are folded over one another (e.g., Figure 1a), so to reduce instrumental volume; the Avaspec bench is non-crossed (e.g., Figure 1b). To enhance signal to noise ratio (SNR), each spectrometer was configured with a cylindrical lens (L2 for the USB2000/2000+; L4 for the USB4000; DCL UV/VIS for the Avaspec) to focus, in the non-dispersive plane, the ≈ 1 mm high incident light beam onto the considerably shorter (≤ 200 μm) detector elements. The detectors were also fitted with coatings/windows in order to increase their sensitivity to UV radiation (UV2, UV4 and DUV, respectively). Data collection and powering of the spectrometers were achieved via USB connection to laptop computers (USB1.1 for the USB2000s and USB2.0 for the others).

The devices were each configured with spectral resolutions ≈ 0.65 nm, as verified by room temperature full width at half maximum measurements of lines from a mercury-argon lamp source (Ocean Optics HG-1). This provides enough detail to adequately capture the SO_2_ absorption spectrum, yet is not so fine as to reduce light levels unnecessarily (e.g., resolution is improved by reducing the width of the entrance slit, and/or using gratings with greater lines/mm, which are more dispersive, yet have poorer light transmission characteristics). The Avaspec optical bench provides fundamentally higher resolution, for a given slit width and grating, than these OO spectrometers. In the former case, incident monochromatic light forms an image at the detector plane approximately as wide as the input slit, as the optical bench curved mirrors are of equal (75 mm) focal lengths; in the latter devices, these mirrors are 42 mm collimating and 68 mm imaging, rendering the image ≈ 68/42 times thicker than the slit width, leading to larger linewidths. For instance, for a 50 μm entrance slit, and a 600 lines/mm grating the Avaspec provides a UV resolution of ≈ 1.2 nm, cf. ≈ 2 nm from the OO devices. In consequence, realisation of the necessary resolution demands a less dispersive grating (1200 lines/mm; UC) for the Avaspec, than for the OO units (2400 lines/mm; #7), resulting in a spectral range of ≈ 210-480 nm for the former spectrometer, and ≈ 250-400 nm for the latter.

The USB2000, USB2000+ and Avaspec all had 2048 element CCD array detectors: the Sony ILX511A and B, respectively in the first two cases, and the Sony ILX554B in the latter; the USB4000's detector was the 3648 pixels Toshiba TCD1304AP (note that in these cases, spectral resolution is defined by the bench, slit width and grating alone; the additional USB4000 pixels do not provide any enhancement in linewidth). The readout electronics provided 16 bit digitization in all cases, save the USB2000, which was 12 bit, with maximum spectral capture rates ranging ≈ 80 - 1,000 Hz between the units. Given that individual spectral exposure times are typically > 100 ms these rates are all more than is required for this application. In respect of dimensions and weight, the OO spectrometers were ≈ 89 × 63 × 34 mm and 190 g, and the Avaspec ≈ 175 × 110 × 44 mm and 720 g, respectively, each compact and field portable, and only a few percent of the mass and bulk of the correlation spectrometers. Similarly, the power requirements (≈ 0.5 W for the USB2000; 1.25 W for the USB4000 and USB2000+; 1.75 W for the Avaspec) are each relatively modest in comparison with the typical (≈ 20 W) consumption of a laptop computer.

## 3. Measurement Accuracy

Provided that a spectrometer has appropriate resolution, and sufficient pixels to meaningfully capture the target species absorption profile (criteria satisfied for all the devices in this study), its utility in atmospheric spectroscopy is determined, to a large extent, by SNR. Signal is defined here by the sensitivity of each detector pixel to incident light at the wavelength it has been tuned to, e.g., according to the angular alignment of the diffraction grating. Noise arises from: 1) the spectrometer's detector and electronics registering a signal even in the absence of incident light: a signature caused, for instance by thermal effects, and which will be superimposed on top of the observed light signal; and 2) stray light from other wavelengths falling upon the pixel, following internal reflections within the spectrometer, e.g., due to imperfections in the optics.

**Figure 2.**
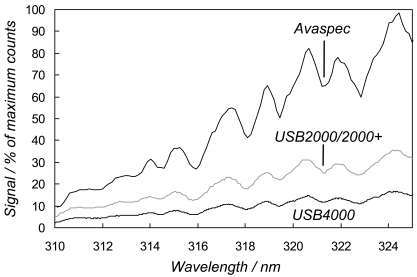
Skylight spectra for simultaneous identical exposure time (170 ms) acquisitions from the Avaspec, USB4000 and a typical USB2000/2000+ spectrometer (USB2+E001).

In order to characterise these phenomena, in respect of our application, we made a series of skylight spectral observations, on February 28^th^ 2008 in Sheffield, under stable intensity clear sky conditions, by coupling the spectrometers via optical fibres (Avantes FCRL-4UV200-2-SR) to co-aligned, home made, single plano-convex f = 100 mm lens, vertically pointing telescopes. Sample 170 ms exposure time spectra for the Avaspec, USB4000 and one USB2000+ are shown in Figure 2, the latter as a representative of the rather similar performance of all the USB2000/2000+ units. The Avaspec displayed higher sensitivity in the ≈ 310-325 nm spectral region, used for the SO_2_ measurement, than the OO devices, with the USB2000/USB2000+s returning around double the signal of the USB4000. This is likely caused, to a large extent, by: 1) the fundamentally higher resolution of the Avaspec optical bench, which allows use of a diffraction grating of fewer lines/mm to realize the desired ≈ 0.65 nm resolution, so providing enhanced light throughput characteristics; and 2) the splitting of the incident light into 3648 pixel increments, in the case of the USB4000's detector, each of correspondingly lower intensity than captured by the other units' 2048 element detectors. These sensitivity data are further illustrated in Table 1, which shows the light signals recorded at 320 nm from each spectrometer. Note that due to the relatively low cost of these units, the performance in each evaluation, per spectrometer type, though broadly similar, would not be expected to be absolutely identical.

We then compared these intensities against the first noise source: the random component of the spectrometer signal in the absence of incident light: the so called “dark current”. This was characterised by observing pixel intensities < 290 nm, where atmospheric ozone completely absorbs down welling UV radiation. The average standard deviation of these pixels, computed from thirty sky spectra were determined, then the mean 320 nm spectral skylight signal divided by this, providing, per spectrometer, a measure of dark current related SNR, as shown in Table 1. Note that the other noise source affecting these pixels: stray light, produced a constant, rather than random signal per pixel under these stable illumination conditions, thus did not perturb the results. Further, by subsequently blocking the spectrometer we verified that the standard deviation of the dark spectra for these pixels is the same, e.g., representative of that at the point of interest, as in the slightly longer wavelength SO_2_ spectral measurement region. The Avaspec ratio (470) is larger than those of the USB2000/USB2000+s (160-295), and the USB4000 (140), mostly due to differences in light sensitivity, rather than dark current noise, the latter being rather similar in each case. Note that these devices also have an additional “offset” component to the no light signal, which is quasi-constant across the pixel range, and ought not to change drastically from spectrum to spectrum, so potentially creating errors. There are, however, longer term thermally related circumstances, whereby this can alter, as discussed in section 4.

The second, stray light, noise source was studied by blocking light from entering the spectrometer and noting to what extent the pixel counts of the skylight spectra < 290 nm were diminished. Any such disparity in light signal could only be caused by scattering of photons of longer wavelengths within the spectrometer, due to the total atmospheric absorption of radiation by ozone in this spectral region. The average pixel drop value was then divided by the spectral intensity at 320 nm, to provide an assessment of stray light related signal to noise for each spectrometer, with results shown in Table 1. Whilst this is a rather crude way of characterising stray light (which, per wavelength has a response that is roughly constant across the spectral domain, except where it rises rapidly in the vicinity of the true wavelength pixel), the quasi-constant reductions observed, per spectrometer, for each pixel in its < 290 nm range, serve to illustrate the better stray light performance of the Avaspec optical bench (ratio of 0.012) versus those of the OO spectrometers (0.023-0.12), in this study. Stray light suppression in the former device is likely expedited by the internal light baffles, aside the beam path within the uncrossed bench design, acting as mitigants against misaligned photons reaching the detector.

In order to characterise the effect of these noise sources on SO_2_ concentration measurements, we placed quartz calibration cells (Resonance Ltd.) of 400 and 1600 ppmm SO_2_ concentration, in front of the telescopes, so as to completely cover their front lenses, and recorded 25 zenith skylight spectra (each obtained by co-adding four 170 ms individual spectra), per spectrometer, per cell. The manufacturer's error budget for these cell concentrations were ≈ ± 50 ppmm and ≈ ± 100 ppmm, respectively, based largely on the uncertainty on other in-house gas cell concentrations, used in the calibration process. For each spectrometer we also recorded a reference spectrum, without the cell in the optical path, and a dark spectrum, with the telescope blocked, according to the same acquisition parameters. The cell spectra were evaluated for SO_2_ concentrations using a differential optical absorption spectroscopy retrieval routine, as is standard in volcanic USB2000 measurements; the particular code was that at the heart of the volcanoSO2.exe program [5]. This, and all other routines used in the forthcoming analyses were written in LabVIEW. Firstly, the dark spectrum was subtracted from each cell spectrum, and from the reference spectrum, the latter spectrum was then divided by the cell spectrum of interest. The natural logarithm was taken, so generating the absorbance spectrum, before high pass filtering, to isolate the rapidly varying component of the SO_2_ absorption spectrum (the so called differential absorption spectrum). Finally, a laboratory standard absorbance spectrum, corresponding to 1 ppmm SO_2_, which had been convolved to match the instrumental spectral resolution, and itself identically high pass filtered (so forming the differential laboratory SO_2_ spectrum), was linearly least squared fitted to the differential spectrum in the 310-325 spectral range, to output the measured concentration.

The mean concentration and standard deviation for the spectra collected with each cell, per spectrometer are shown in Table 1; the former data for the Avaspec (392 and 1644 ppmm, respectively) more closely resemble the true cell concentrations than those from the OO devices (ranging 332-384 and 991-1421 ppmm). Indeed, none of the non-Avaspec spectrometers measure the 1600 ppmm cell concentrations within the measurement error. Furthermore, the order of the mean concentrations is almost exactly opposite to that of the stray light / 320 nm signal values. Stray light essentially acts to reduce the contrast between the cell and reference spectra, caused by the SO_2_ absorption, by adding counts to each, which are not removed by subtraction of the dark spectrum, so decreasing apparent absorbance, particularly at the shortest wavelengths, where light levels are weakest. Hence: more stray light, lower retrieved concentrations.

Stray light can be computationally compensated for, to some degree, by monitoring the lowest pixel count in each dark subtracted sky spectrum, and subtracting that from all other pixels in that spectrum, prior to the next analysis stages. This of course is rather crude, given that the stray light signature is not spectrally flat, rather elevated across the pixels corresponding to the wavelengths of the incident skylight. Nonetheless, where this is applied, this does cause the lower spectrometer concentrations to rise, in most cases to be within error of the cell column amounts, as documented in Table 1. By reducing the systematic error in this way, however, the measurement random error is increased, as expressed in the concentration standard deviations, as the stray light had acted to suppress dark current generated inter-spectra differences. This is particularly obvious in the case of the most stray light prone of the analysed units: USB2G2385.

Alternate stray light mitigative strategies include using a low pass filter to block visible light from entering the spectrometer; however this does not deal with stray light originating from the UV. Similarly, increasing the fit window start wavelength so to exclude the most adversely affected pixels could in principle raise the lower retrieved concentrations somewhat. However, this would reduce the number of data points available for fitting, increasing the measurement random error. A recently proposed alternate of measuring the instrumental stray light response function using laser line sources, enabling skylight spectra to be corrected accordingly, shows promise for further error reduction in this regard [17].

**Figure 3.**
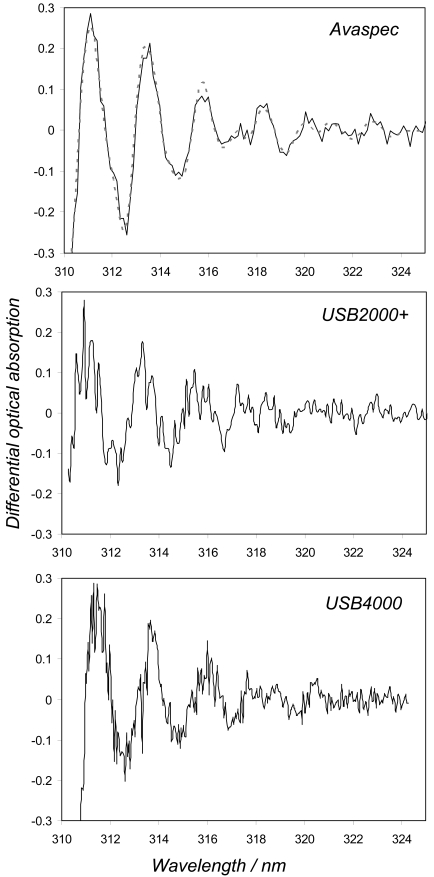
Differential absorption spectra, obtained from the 170 ms exposure time 1600 ppmm SO_2_ cell measurements with the Avaspec, USB2+E001, and the USB4000, in the former case providing the best visual match to the scaled laboratory absorbance spectrum (dashed line in top plot).

From the perspective of random error in the cell spectral measurements, the Avantes, as with respect to systematic errors, performed the best of the analysed spectrometers in this study (e.g., concentration standard deviations of 29 and 16 for the 1600 and 400 ppmm cells, respectively vs. 50-78 and 24-43 for the OO spectrometers, for the stray light corrected data) as a result of its higher light sensitivity/dark current noise characteristics (Table 1). Indeed, it is clear from the differential absorption spectra shown in Figure 3, corresponding to 1600 ppmm cell measurements, taken with the Avaspec, USB4000 and USB2+E001 (taken as a typical representative of USB2000/USB2000+ behaviour), that the former data are less noisy. These experiments were deliberately performed with identical exposure and data evaluation procedures in order to provide the most equivalent inter-comparisons. However, due to the better stray light characteristics of the Avaspec, the start pixel of the fit window could be reduced here, with no drop off in retrieved concentrations, further improving upon its standard deviations, by including more pixels in the fitting.

The one exception to the better standard deviation performance of the Avaspec, are the non stray light corrected values for USB2G2385. These are artificially low, however as the stray light suppresses inter-spectrometer differences, at the expense of imposing large systematic errors. Once stray light is corrected for, these standard deviations become more representative of those of the other OO spectrometers, as seen in Table 1. Whilst the USB4000 had lower dark current related signal to noise characteristics to those of the other OO spectrometers, its concentration standard deviations were similar, likely due to the compensating effect of the greater number of pixels involved in the fitting.

## 4. Thermal Effects

The previous section concerns the spectrometers' accuracy in making SO_2_ concentration measurements over relatively short periods in near room temperature conditions (≈ 18 °C). Over longer periods (e.g., that of a traverse; 10s of minutes), and/or across different environments, the range of encountered thermal conditions may vary considerably, however, with a number of potentially important implications for sensor performance. The first such effect concerns the offset, e.g., the component of the no-light signal, which is quasi-constant across the pixel range, and is modulated by temperature, so shifting the superimposed dark current and light signal spectra up or down. We assessed this, firstly, by simply switching the spectrometers on and, as the units warmed up, monitoring trends in offset values, e.g., the average pixel value per spectrum (300 ms acquisition), with light blocked from entering the spectrometer. Note that in this experiment this drift was the only observed sensor change perceived to be capable of perturbing retrieved SO_2_ signals. Each spectrometer stabilised after ≈ 30 minutes, during which time the USB4000 offset increased by 25%, the Avaspec by 10% and the USB2000s and USB2000+s decreased by 38% and 34%, respectively.

We secondly placed the spectrometers in a temperature stabilised (± 0.5 °C) incubator unit (LMS ltd. series 3, 400W), and examined the offset variation in heating from 12 °C to 32 °C, in ≈ 7 °C increments. In accord with the previous experiment, the offset rose for the USB4000 (≈ 120 %) and for the Avaspec (≈ 70%), and fell for the USB2000s and USB2000+s (≈ 100%) in each case at quasi-constant rates. For the largest of the spectrometers' offset change rates, it is conceivable that an ambient temperature change of a few degrees, and/or spectrometer warm up, could cause offset shift of > ± 10% during individual traverse SO_2_ flux measurements. The effect of this on concentrations was simulated by vertically shifting recorded cell spectra, then evaluating them with respect to un-shifted reference spectra. The retrieved concentrations differed from those determined with no such shift by > ± 10%.

These offset errors can be readily eliminated, to a large extent, however, by simply monitoring the upward or downward modulation of the short wavelength pixels, where ozone absorbs down welling skylight, and shifting the whole spectrum up or down accordingly, so to fix the offset. The one exception to this is when the offset shifts to a negative value, in which case the no-light signal simply returns zero values. The major implication of this is that it also draws down the observed light spectra, perhaps more so than the response to light of the shortest wavelength skylight pixels can counteract, so that these elements register zero counts. This spectral truncation creates a series of computational problems for the SO_2_ retrieval, not least a shortening of the pixel range, available for the spectral fitting, potentially leading to significant errors. It is highly desirable to avoid negative offset, therefore, for instance through thermal stabilisation of the spectrometer, or use of the Ocean Optics USB EEPROM programmer. During our experiments, negative offset was just starting to occur for one of the USB2000s and one of the USB2000+s at 32 °C, with the other USB2000s and USB2000+ no light signals hovering just above zero counts. The Avaspec and USB4000 had no such problems in our studied 12 °C to 32 °C temperature range, although, given their positive correlations between temperature and offset, they may demonstrate negative offset at lower temperatures.

During the incubator experiments we also studied the variation in spectral alignment, sensitivity and resolution in heating from 12 °C to 32 °C, by taking fibre coupled measurements of lines (296.7 and 302.1 nm) from the mercury-argon lamp source, in the vicinity of the SO_2_ absorption spectrum fit window. In respect of the first of these phenomena, these lines positions varied very little with temperature (typically ≤ 1 pixel) for all the spectrometers, ruling this out as a significant source of error, at least for these units. For the second study, the sensitivity changed by ≤ ≈ 10% in each case. This too is unlikely to have any great bearing on retrieved concentrations, however, as it essentially corresponds to multiplication of the skylight signals by a scalar, which will appear as a constant after the logarithm stage of the evaluation, becoming eliminated by the high pass filter.

**Figure 4.**
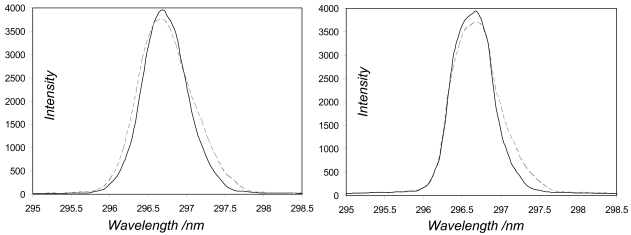
Thermal variation in the profile of the 296.7 nm line in the Hg lamp experiments. Traces at 12 °C are dashed, and solid for 32 °C, showing heating induced line narrowing from ≈ 0.8 nm to 0.65 nm for USB2G1110 (left) and from ≈ 0.7 nm to 0.6 nm for USB2G2385 (right).

The last of these sensor characteristics, namely the resolution, was addressed by monitoring the full width at half maximum of the lamp lines as the spectrometers were heated, e.g. how broadened do these devices make these infinitesimally narrow features appear. For all the spectrometers, save two of the USB2000s (USB2G1110 and USB2G2385), the linewidth varied insignificantly (< 0.05 nm) across the temperature range. For these exceptions, the lines' spectral widths narrowed by ≈ 0.15 and 0.1 nm, respectively, likely due to thermal expansion of optical bench components with increased temperature (e.g., Figure 4). This would act to increase the amplitudes of the peaks and troughs in the observed SO_2_ differential absorption spectra (e.g., see Figure 3), for a given observed column amount, which could be readily misinterpreted as an increase in concentration. Whilst a 20 °C change in temperature is highly unlikely to occur over an individual traverse, and the changes to linewidth associated with the more plausible ≤ few degree shifts are insignificant for these USB2000s, an issue is raised as to how these units, if calibrated at room temperature, would perform in a far cooler or warmer environment (e.g., Erebus volcano in Antarctica or equatorial Nyiragongo volcano). In practice, linewidth calibration is achieved by measuring the profile of a mercury line with the candidate spectrometer, then convolving this with a very high resolution standard SO_2_ absorption spectrum, to degrade the latter to the instrument's rather poorer resolution. A high pass filter is then applied to form the differential laboratory SO_2_ spectrum, used in fitting to the observed field differential absorption spectra. We investigated the implications of the most extreme of these USB2000 thermally related lineshape changes by generating two differential laboratory SO_2_ spectra, with the USB2G1110 302.1 nm line shapes measured at 12 °C and 32 °C, respectively (≈ 0.8 nm and 0.65 nm widths). The former spectrum was then least squared fitted to the latter, which was thereby determined to have ≈ 12% larger peak/trough amplitudes; e.g., use of the differential laboratory spectrum generated at 12 °C to evaluate field spectra collected at 32 °C would result in a corresponding concentration overestimation, by failing to compensate for narrowing in instrumental linewidth, which results in a sharpening of observed spectral features. It remains unclear whether investigation of further non-USB2000 OO spectrometers or Avaspecs would reveal similar thermal linewidth behaviour. All these thermal results issue a caution, therefore against indiscriminate usage of any of these compact spectrometers without consideration of extrinsic factors, such as changes in temperature, which could significantly perturb performance, and for which reason a number of workers thermally stabilise their spectrometers [12].

## 5. Conclusions and Recommendations

In respect of the side by side measurement accuracy studies, with identical spectral acquisition parameters, the Avaspec demonstrated the best performance; whilst this unit is highly field portable, it is larger and more power consuming than the other studied devices, however (e.g., 710 g and 1.75 W vs. 190 g and 1.25 W for the Avaspec and USB2000+, respectively). The primary advantage of the Avaspec here is its high optical sensitivity bench, which demands relatively short exposure times to near saturate the spectrometer in the spectral fitting window, so to maximise these pixels' SNRs. This would be particularly useful where the highest temporal resolutions are required, e.g., when traversing by plane a narrow plume, or studying Strombolian explosions, which last but a few seconds. In other scenarios, e.g., probably for the vast majority of cases, where longer exposure times are tolerable, so that the OO spectrometers can saturate in the fitting window, the SNRs of the OO units will improve correspondingly, and the units behaved very similarly to the Avaspec. For instance, side by side SO_2_ cell tests between the Avaspec and USB2G3709, with both units near saturated by operating on different integration times, revealed near identical standard deviations, and within error concentration estimations, with stray light correction, in both cases.

The USB2000+ and the USB4000 performed rather similarly to the USB2000. As the studied USB4000 spectra demonstrated spurious structures on occasion in the seconds following a change in integration time, our preference was for the USB2000+s. Hence our recommendations are: in situations (probably covering most cases) where there is no motivation to acquire SO_2_ data faster than has already been demonstrated possible with the USB2000s, and where there is a desire to maintain the ultra-compact footprint of this device and minimise power budgets, the USB2000+ represents an ideal technology for future volcanic gas measurements; however, where very rapid acquisitions are required, users may consider the Avaspec, as a similarly priced alternate, albeit with greater bulk and power requirements. We further reiterate the importance of being aware of, and appropriately managing, the aforementioned thermal issues, to ensure their effects upon measurement accuracy are minimised. In view of the two possible instrumental routes that users may wish to take forward, depending on their measurement requirements, we are in the process of finalizing a user friendly software interface, which we anticipate making freely downloadable, to enable volcanic SO_2_ flux measurements to be made with either the Avaspec or USB2000+. This code will also work for the USB2000, and provides improved measurement accuracy, relative to its predecessor code: VolcanoSO2.exe [5].

The spectrometers covered in this study represent a non-exhaustive list of those currently available in a similar price band to the original USB2000, and serve to illustrate the point that performance is enhanced with improved SNR. OO have also released a larger and higher sensitivity version of the USB2000+, the HR2000+, which, with an appropriately chosen grating, may well equal, or even exceed the Avaspec in performance. This unit retailed in a higher price band than the units included in our study, so was not considered here, however. Another route to yet greater performance, comes via the recent introduction of back thinned CCD array detectors, now implemented in a number of market available spectrometers, providing an additional means of improving SNR, yet, again at greater cost (> €4,000). Finally, optional, and relatively inexpensive upgrades for both the Avaspec and USB2000+, in respect of the detector in the former, and grating in the latter, have very recently become available, with purported improvements in light sensitivity in both cases.

## Figures and Tables

**Table 1.** Summary of the results of the investigations into the spectrometer characteristics reported in this paper for 170 ms integration time sky spectra. Full detail is supplied in the text.

Spectrometer	USB2000: G3709	USB2000: G1110	USB2000: G2385	USB4000: C0004	USB2000+: E001	USB2000+: F002	Avaspec: 0802095U1
320 nm skylight signal (% of Avaspec value)	42%	38%	55%	19%	42%	34%	100%
320 nm signal / dark current random noise	295	250	220	140	275	160	470
Stray light / 320 nm signal	0.031	0.038	0.12	0.023	0.050	0.069	0.012
Measured concentration 1600 cell	1373 ± 42 ppmm	1421 ± 41 ppmm	991 ± 22 ppmm	1382 ± 49 ppmm	1171 ± 39 ppmm	1126 ± 52 ppmm	1644 ± 28 ppmm
Measured concentration 400 cell	362 ± 36 ppmm	384 ± 30 ppmm	338 ± 14 ppmm	371 ± 26 ppmm	356 ± 23 ppmm	332 ± 30 ppmm	392 ± 16 ppmm
Stray light corrected concentrations 1600 cell	1595 ± 54 ppmm	1643 ± 53 ppmm	1540 ± 50 ppmm	1422 ± 51 ppmm	1542 ± 66 ppmm	1550 ± 78 ppmm	1679 ± 29 ppmm
Stray light corrected concentrations 400 cell	395 ± 42 ppmm	411 ± 35 ppmm	417 ± 24 ppmm	375 ± 27 ppmm	408 ± 27 ppmm	411 ± 43 ppmm	396 ± 16 ppmm
